# Genetics, sex and the use of platelet‐rich plasma influence the development of arthrofibrosis after anterior cruciate ligament reconstruction

**DOI:** 10.1002/jeo2.70156

**Published:** 2025-01-28

**Authors:** Mikel Sánchez, Izarbe Yarza, Cristina Jorquera, Jose María Aznar, Leonor López de Dicastillo, Cristina Valente, Renato Andrade, João Espregueira‐Mendes, David Celorrio, Beatriz Aizpurua, Juan Azofra, Diego Delgado

**Affiliations:** ^1^ Arthroscopic Surgery Unit Hospital Vithas Vitoria Vitoria‐Gasteiz Spain; ^2^ Advanced Biological Therapy Unit Hospital Vithas Vitoria Vitoria‐Gasteiz Spain; ^3^ Baigene Vitoria‐Gasteiz Spain; ^4^ Clínica Espregueira‐FIFA Medical Centre of Excellence Porto Portugal; ^5^ Dom Henrique Research Centre Porto Portugal; ^6^ Porto Biomechanics Laboratory (LABIOMEP) Faculty of Sports, University of Porto Porto Portugal; ^7^ School of Medicine University of Minho Braga Portugal; ^8^ ICVS/3B's–PT Government Associate Laboratory Braga/Guimarães Portugal; ^9^ 3B's Research Group—Biomaterials, Biodegradables and Biomimetics, Headquarters of the European Institute of Excellence on Tissue Engineering and Regenerative Medicine University of Minho Braga/Guimarães Portugal

**Keywords:** algorithm, anterior cruciate ligament, arthrofibrosis, genetics

## Abstract

**Purpose:**

To identify genes and patient factors that are related to the development of arthrofibrosis in patients after anterior cruciate ligament (ACL) reconstruction and to develop a prognostic model.

**Methods:**

The study included patients diagnosed with ACL injury who underwent ACL reconstruction. Patients were enroled consecutively and divided into non‐fibrotic (controls) and fibrotic (cases) groups until a balanced sample of matched case–control was achieved. Arthrofibrosis was considered pathological if the range of motion achieved 3 months after surgery decreased by at least 25% compared to its initial full range of motion. Patient variables and saliva samples were collected from each patient to perform a genetic approach by screening a set of candidate genes implicated in arthrofibrosis. Chi‐squared was used to analyze the association between the development of arthrofibrosis and different independent variables. Binary logistic regression was used to develop a prognostic algorithm.

**Results:**

A total of 45 controls (non‐fibrotic patients) (50.1%) and 44 cases (fibrotic patients) (49.9%) were included for analysis. The median age was 34.0 years (95% confidence interval = 29.0–38.0) and the number of women was 32 (35.9%). Seven genetic polymorphisms showed significant association with the development of arthrofibrosis (*p* < 0.05). After binary regression analysis, the regression model included the polymorphisms rs4343 (ACE), rs1800947 (CRP), rs8032158 (NEDD4) and rs679620 (MMP3). This analysis also indicated that female gender was a risk factor while the use of platelet‐rich plasma (PRP) during surgery was a preventive factor (*p* < 0.05).

**Conclusion:**

Genetic alterations involved in inflammation and extracellular matrix turnover predispose to the development of arthrofibrosis after ACL reconstruction. Female sex was a risk factor in the development of this condition, while the application of PRP provided a preventive effect. The combination of patient and genetic variants of a patient allows the development of a prognostic algorithm for the risk of post‐surgical arthrofibrosis.

**Level of Evidence:**

level III.

**Abbreviations**
A: adenine
ACE: angiotensin‐converting enzyme
ACL: anterior cruciate ligament
AQP1: aquaporin 1
AUC: area under the curve
BMI: body mass index
C: cytosine
CI: confidence interval
CRP: C‐reactive protein
CTGF: connective tissue growth factor
D: deletion
G: guanine
I: insertion
IL1: interleukin 1
IL6: interleukin 6
MMP3: matrix metallopeptidase 3
NEDD4: E3 ubiquitin protein ligase
NF‐kβ: Nuclear factor kappa‐light‐chain‐enhancer of activated B cells
OR: odds ratio
PAI‐1: plasminogen activator inhibitor 1
PRP: platelet‐rich plasma
SOD2: superoxide dismutase 2
T: thymine
TGF‐β: transforming growth factor beta
TNF: tumour necrosis factor

## INTRODUCTION

Anterior cruciate ligament (ACL) rupture is a common sports injury, and its reconstruction is one of the most common treatments. Despite its positive clinical outcomes, it still presents challenges that need to be addressed such as the risk of complications arising from ACL reconstruction, including most notably arthrofibrosis. In fact, in patients who underwent knee surgery, the prevalence of arthrofibrosis ranges from 1% to 15% [16] and increases especially in the case of ACL reconstructions, varying from 4% to 38% [8].

Although still poorly defined, arthrofibrosis can be described as the loss of flexion and/or extension of the joint or also as a pathological process in which fibrotic tissue grows in an uncontrolled manner [8, 12]. The pathophysiology of arthrofibrosis is highly related to inflammation‐generating cytokines and molecules such as transforming growth factor beta (TGF‐β) that promote the proliferation of myofibroblasts not usually found in healthy tissues [42]. As a consequence, these patients suffer from pain and reduced mobility, which hampers their post‐operative clinical evolution and knee function [26].

Prevention or early detection thus plays a key role in the management of arthrofibrosis, and it is the focus of the efforts of the medical‐scientific community [8, 20].

Recent studies focus on the analysis of variables to estimate a patient's risk factors. These factors include age, sex or surgical techniques [14, 35]. In recent years there has been an emerging and growing interest in the role that genetics may play in the development of this arthrofibrosis [3, 28]. More specifically, individuals who developed arthrofibrosis have a family history of the same pathology, suggesting the intervention of genetic factors in its development [36]. In a recent study assessing the risk of female basketball players developing arthrofibrosis, the importance of ethnicity was also demonstrated [7]. This suggests that genetics is a factor to be taken into account in the possible prediction.

Understanding how genetic and patient factors can trigger the arthrofibrosis process offers the opportunity to develop potential strategies to prevent or modulate the development of arthrofibrosis. However, there is still some way to go to fully unravel the underlying mechanisms behind these genetic associations and their clinical implications.

The main objective is to identify genes and patient factors related to the development of arthrofibrosis after ACL reconstruction and to develop a prognostic model. This work is based on the hypothesis of being able to know and quantify the influence that genetic and patient factors have on the appearance of arthrofibrosis. This would make it possible to predict its development and thus give healthcare professionals the opportunity to apply strategies for its prevention.

## METHODS

### Patients and study design

The study was designed as a prospective consecutive case‐control study to analyze the influence of genetic and patient factors on the development of arthrofibrosis following ACL reconstruction. This study was carried out in accordance with the International Declaration of Helsinki (Fortaleza, Brazil; 2013) and Good Clinical Practice. Ethical approval (protocol no: UCA‐07/EE/17/FIB) was obtained by the OSI Araba Ethics Committee (June 2017), and informed consent was obtained from patients. The eligible patients were enroled consecutively between 2017 and 2021 in the same medical centre until a balanced sample of matched case–control was achieved. All participants met the following inclusion criteria: patients of both sexes over 18 years old and diagnosed with ACL injury according to clinical findings and image studies who underwent ACL reconstruction. Patients operated on in the inflammatory phase, without complete range of motion, having osteochondral and multiligament pathology, as well as revision cases were not eligible for the study. Patients were divided into non‐fibrotic (controls) and fibrotic (cases) groups based on the development of arthrofibrosis.

### Surgical technique

Patients underwent surgery at least 1 month after the injury and after the inflammatory phase had resolved. Prior to surgery, patients underwent physical exercise to reduce inflammation, achieve proper muscle activation and improve mobility in order to reach full joint balance (0–120/130°). All patients underwent notchplasty after cleaning the ACL remnants for three main reasons: to prevent future graft impingement (1), correct visualization and placement of the graft in the femoral tunnel (2), and to increase the cellularity of the area to improve graft integration (3). Once the joint site was prepared, the hamstring autograft was obtained.

The bone tunnels were performed and the fixation of the graft to the femur was performed using the TightRope cortical implant (Arthrex). Tibial fixation was performed by means of bone plugs removed during bone tunnelling in addition to the use of two fixation staples. The bone grafts were reimplanted after graft positioning and the tibial tunnel was sealed, providing biological fixation (Figure S1). The total duration of this surgery is approximately 60 min.

In patients who agreed to, platelet‐rich plasma (PRP) was used as biological augmentation at the different steps, including the infiltration and soaking of the grafts and bone plugs, the intraosseous infiltration of the tunnels and surroundings, and intra‐articular injection as a final step. The PRP used (BTI Biotechnology Institute) contained a moderate concentration of platelets (about two times blood platelet levels), with no erythrocytes or leucocytes and calcium‐activated [39]. According to the UCS for PRP studies, this PRP is usually 13‐00‐11 [18].

### Post‐operative protocol and recommendations

During the first 14 days following surgery, patients were instructed to rest, with the knee extended, and apply ice on a regular basis to reduce inflammation and pain. However, on the second day after surgery, patients were instructed to perform simple strength and mobility exercises removing the immobilizer. On the 10th day after surgery, rehabilitation was initiated in a progressive and pain‐free manner. Knee flexion should not exceed 90° during the first 4–6 weeks after surgery. During these weeks, the immobilizer was maintained when walking and as protection, but it was removed during the rehabilitation exercises, with partial weight bearing. Crutches were removed progressively, being mandatory during the first 4 weeks, depending on the involvement of concomitant interventions such as meniscal repair.

The rehabilitation process consisted of static strengthening associated with electrostimulation from the beginning of recovery to strengthen the hamstrings and quadriceps muscles. Once the crutches have been removed, active strengthening exercises begin by performing concentric work on the quadriceps in a closed kinetic chain. After 3 months, open kinetic chain exercises were introduced. Concentric exercises to strengthen the hamstrings were implemented with progressive loads. Gluteal muscle strength training and progressive proprioception exercises were also implemented during the rehabilitation process.

### Outcome evaluation

The evaluation of arthrofibrosis was dichotomized (presence: yes/no) according to the degree of mobility or joint balance. It was considered pathological (yes) if the range of motion achieved 3 months after surgery decreased by at least 25% compared to its initial full range of motion (0–120°/130°) not reaching 100° of flexion, and/or if adhesions were present requiring manipulation under anaesthesia and/or lysis of adhesions. The patient variables collected at baseline were age, sex, blood pressure [31], body mass index (BMI) and application of PRP in surgery.

### Genotyping

Saliva samples for DNA analysis were obtained using 4N6FLOQSwabs (Life Technologies). DNA was extracted using the QIAmp DNA Mini kit (Qiagen), and it was quantified fluorometrically by Qubit (Life Technologies). Samples were genotyped using the Biomark HD System Technology (Fluidigm).

A total of 34 polymorphisms involved in keloid formation, tissue fibrosis, tissue growth, scarring, cell damage, inflammation and cell regeneration were selected for the genotyping in this study (Table S1).

### Statistical analyses

The IBM SPSS Statistics (version 25) software was used to determine an arthrofibrosis prognostic model and R software version 4.1.1 (2021‐08‐10) SNPassoc package was used to analyze the association between the development of arthrofibrosis and the different genetic polymorphisms under five different genetic models (inheritance patterns): codominant, dominant, recessive, over dominant and log‐additive models and Hardy–Weinberg equilibrium (HWE) test [13]. Chi‐squared test was used to analyze the relationship between the development of arthrofibrosis and independent variables. Binary logistic regression was used to calculate the odds ratio (OR) with a 95% confidence interval (CI) of developing arthrofibrosis. The area under the curve (AUC), Hosmer and Lemeshow test and the Nagelkerke R‐squared test were calculated for goodness of fit of the model [6]. A value of (1 − *β*) = 0.9977309 was obtained from the post hoc power analysis test performed using version 3.1G*Power software taking into account the number of predictors, the arthrofibrosis sample size and the *R* ^2^ value obtained from the model.

## RESULTS

### Demographics and patient characteristics

The study included a total of 89 patients (Table 1). The median age was 34.0 years (95% CI = 29.0–38.0), with a median BMI of 23.2 (95% CI = 22.5–24.3) and 32 women (35.9%). Table 1 shows the characteristics of the patients classified into controls and cases.

**Table 1:** Demographic and clinical characteristics.

Parameter	No arthrofibrosis (controls)	Arthrofibrosis (cases)	*p*
*N*, count	45	44	
Age, median (95% CI)	31 (27–40)	35 (31–38)	*n.s*.
BMI, median (95% CI)	22.6 (21.7–24.1)	23.9 (22.7–25.6)	*n.s*.
Female, count (%)	12 (26.7)	20 (45.4)	*n.s*.
High blood pressure, count (%)	4 (8.9%)	1 (2.3%)	*n.s*.
PRP, count (%)	43 (95.6%)	17 (38.7%)	<*0.001*
Meniscal intervention, count (%)	23 (51.1%)	19 (43.2%)	*n.s*.

Abbreviations: BMI, body max index; CI, confidence interval; n.s., not significant; PRP, platelet‐rich plasma.

### Association analysis between arthrofibrosis and independent variables

Of the patient factors analyzed, only the use of PRP showed a highly significant association with the prevention of arthrofibrosis development (*p* < 0.001).

The results of the Hardy–Weinberg equilibrium test showed that all polymorphisms were in equilibrium except for rs1800947 (CRP gene). Of the screened polymorphisms, only seven revealed an association with arthrofibrosis (Table 2).

**Table 2:** Significant association results between the polymorphisms analysed and arthrofibrosis.

Polymorphism	IM	*p*	AIC	OR	AIC
rs1049305	Recessive	*0.021*	122	1.00	0.09–0.87
				0.28	
rs1800795	Recessive	*0.022*	122.1	1.00	
				3.84	1.13–13.05
rs4880	Dominant	*0.019*	121.8	1.00	
				3.21	1.17–8.79
rs679620	Dominant	*0.012*	121.1	1.00	
				0.32	0.13–0.8
rs9493150	Codominant	*0.026*	122	1.00	
				3.00	1.2 –7.37
				0.67	0.11–3.88
rs1800947	Dominant	*0.015*	121.4	1.00	
				0.32	0.13–0.82
rs1799768	Dominant	*0.009*	120.7	1.00	
				0.3	0.12–0.77

Abbreviations: AIC, Akaike information criterion; CI, confidence interval; IM, inheritance model; OR, odds ratio.

### Prognostic model for the development of arthrofibrosis

After binary regression analysis, a prognostic model was obtained that included the polymorphisms rs4343 (ACE), rs1800947 (CRP), rs8032158 (NEDD4) and rs679620 (MMP3) together with the independent variables sex and PRP administration (Table 3).

**Table 3:** Variable under the prognostic model for development of arthrofibrosis after ACL reconstruction.

Variable	*B*	*p*	OR	95% CI
Constant	−2.070	*n.s*.	0.126	
Sex (women vs. men^a^)	1.859	*0.005*	0.156	0.042–0.577
PRP (no vs. yes^a^)	3.075	*0.001*	21.652	3.386–138.460
ACE (rs4343)		*0.014*		
ACE (rs4343) (1) (D:D vs. I:I^a^)	0.869	*n.s*.	2.383	0.269–21.098
ACE (rs4343) (2) (I:D vs. I:I^a^)	2.596	*0.015*	13.407	1.640–109.631
CRP (rs1800947)		*0.039*		
CRP (rs1800947) (1) (C:G^a^ vs. C:C)	−2.141	*0.013*	8.511	1.577–45.948
CRP (rs1800947) (2) (G:G^a^ vs. C:C)	−1.161	*n.s*.	3.194	0.422–24.147
NEDD4 (rs8032158)		*0.016*		
NEDD4 (rs8032158) (1) (C:T^a^ vs. C:C)	−1.654	*n.s*.	0.191	0.029–1.262
NEDD4 (rs8032158) (2) (T:T vs. C:C^a^)	2.331	*0.005*	0.097	0.019–0.486
MMP3 (rs679620) (1) (A:G‐A:A^a^ vs. G:G)	−1.454	*0.024*	4.280	1.208–15.171

Abbreviations: A, adenine; B, coefficient; C, cytosine; CI, confidence interval; D, deletion; I, insertion; G, guanine; n.s., not significant; OR, odds ratio; PRP, platelet‐rich plasma; T, thymine.

^a^ Protection factor.

**Figure 1:**
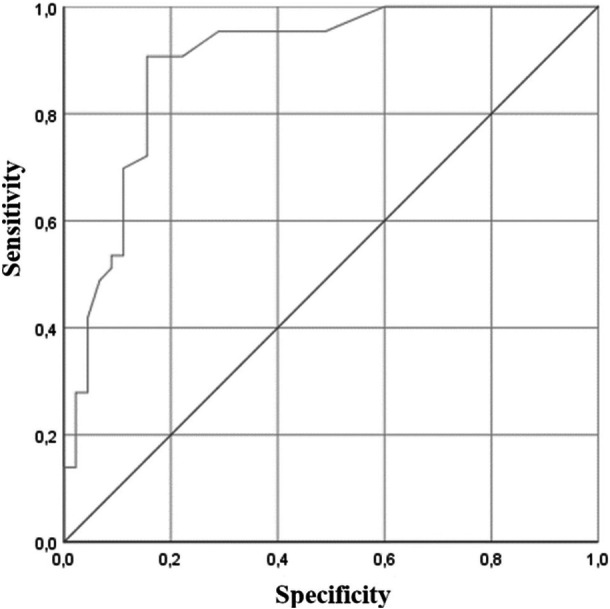
Receiver operating characteristic curve of the prognosis model with an area under the curve of 0.897.

The results of Hosmer and Lemeshow's Chi‐square test yielded a value of *p* = 0.055 and Nagelkerke's *R* ^2^ test yielded a value of *R* = 0.557. An AUC of 0.897 (95% CI = 0.829–0.965) was also obtained (Figure 1).

Table 4 compares the predicted results against what was observed in the case‐control study for the model obtained, indicating the values of sensitivity, specificity and overall success rate.

**Table 4:** Predicted results versus observed results in the model obtained.

		Predicted cases			
		Non‐arthrofibrosis	Arthrofibrosis		
Observed cases	Arthrofibrosis	4	40	Sensitivity	90.9%
	Non‐arthrofibrosis	38	7	Specificity	84.4%
				Accuracy	87.6%

## DISCUSSION

The most important finding of this study was the relationship between genetic variants, patient factors and the development of post‐operative arthrofibrosis after ACL reconstruction, achieving a prognostic model with an ROC curve of 0.897.

Among the patient factors, sex was shown to be the only demographic characteristic of the patients that influenced the development of arthrofibrosis. Female sex was found to affect the risk of arthrofibrosis development by increasing it by 0.6‐fold. These data showing that women had a higher risk of arthrofibrosis are consistent with previous studies [14, 35]. In a recent study conducted by Rahardja et al. [35], women had a 1.8‐fold higher risk of developing arthrofibrosis after ACL reconstruction than men. In a very recent systematic review with meta‐analysis [14], the female sex was also a higher risk factor related to manipulation under anaesthesia and lysis of adhesions. Although the hormonal component may play an important role in these sex differences [42], other biological components may also be involved. Kryczka et al. [19] observed that the level of fibrinogen in blood was higher in women than in men, with a higher presence of fibroblasts, B and T 40 cells, a higher amount of extracellular matrix (ECM) and overexpression of some inflammation‐related genes.

Regardless of the mechanisms of action that trigger fibrosis, inflammation appears to underlie this process [15]. Indeed, several studies have proposed strategies to mitigate fibrosis through anti‐inflammatory approaches [21, 29]. This could explain how in this study the application of PRP during ACL reconstruction reduced the risk of arthrofibrosis by up to 21.7‐fold. This biological product contains a large number of biomolecules, many of which are involved in the inhibition of pro‐inflammatory processes such as brain‐derived neurotrophic factor [25] and hepatocyte growth factor [4, 44]. Thus, one of the main mechanisms of action of PRP is its anti‐inflammatory effect [37]. which could be very valuable in mitigating the inflammatory reactions of injuries and surgical interventions [38], such as ACL reconstruction, reducing the risk of developing arthrofibrosis.

Regarding the genetic impact on the development of arthrofibrosis after ACL reconstruction, among all the genetic variants screened, some were statistically significantly related to the development of fibrosis, including IL6 (rs1800795), SOD2 (rs4880), AQP1 (rs1049305), CTGF (rs9493150), MMP3 (rs679620), CRP (rs1800947) and PAI‐1 (rs1799768). For rs1800947 (CRP gene), the HWE test showed that this polymorphism was not in equilibrium for this population. HWE principle states that genetic variation in a population will remain constant from one generation to the next in the absence of perturbing factors. Deviations from HWE may indicate some type of selection, inbreeding or population stratification. The absence of HWE for this SNP could be due to several reasons, including a limitation in the number of the sample or a possible higher prevalence of this polymorphism in ACL injuries.

However, when the algorithm was developed, only four of the genes analyzed (ACE, CRP, NEDD4 and MMP3) were entered into the prognosis model together with sex and PRP as patient variables. As mentioned above, the fibrosis process has an inflammatory component, with CRP being one of the systemic markers related to inflammation that promotes the production of IL1 and TNF, through a TGF‐β/Smad3 or NF‐ĸB‐dependent mechanism, leading to ECM deposition [45]. The results of this study conclude that the GG genotype for the CRP gene (rs1800947) results in a reduced risk of fibrosis in the absence of an exacerbated inflammatory response [1, 33].

The ACE, MMP3 and NEDD4 genes included in the prediction model are involved in the phases of tissue formation and remodelling. A previous study [27] has shown that ACE activity in pathological scar tissue was greater than in normal skin. Furthermore, the DD genotype in the ACE gene (rs4343) causes overexpression of angiotensin‐converting enzyme [19] and therefore greater development of fibrosis [17]. Thus, according to these studies, the results achieved in this study indicated that II has a protective effect.

As for the MMP3 gene, G allele (rs679620) causes its overexpression, generating scenarios such as an increased risk of tendinopathy [9, 24]. However, a higher risk of these lesions in subjects with the AA genotype has also been reported [5]. The present study revealed that the GG genotype is associated with a higher risk of fibrosis, possibly because overexpression of this gene is related to disorders in the elimination of damaged tissue in injuries and surgeries.

Finally, the NEDD4 gene encodes the E3 ubiquitin ligase protein, which is involved in ubiquitination or modulation of skin fibroblast proliferation, differentiation and collagen secretion. The NEDD4 gene is involved in different points of scar metabolism (SMAD4, NF‐ĸB/STAT3 and TGF‐β1) [11, 40] and it is found in different tissues such as skeletal muscle, bladder, liver and skin [30]. The C allele of NEDD4 (rs8032158) downregulates the concentration of E3 ubiquitin ligase, which increases the risk of fibrosis [10, 32]. In this study, the genotype with the highest risk is TT, which does not coincide with previous literature. There is a general scientific consensus on the relationship of each NEDD4 genotype, with the development of fibrosis [10, 32]. Even the research has been focusing on epithelial tissue (keloid pathway). In this way and concerning the gap in literature about studies which address arthrofibrosis and ligamentous tissue, this study is considered a milestone, which relates NEDD4 (rs8032158) to this pathology.

Other genes showed statistical significance but were not included in the model. Interleukin 6 (IL6) (rs1800795) related to inflammation in the early stages of scarring [41, 43], SOD2 (rs4880) related to autophagy through sirtuin 3 [23], AQP1 (rs1049305) which attenuates fibrosis through regulation of epithelial–mesenchymal transition [22], CTGF (rs9493150) whose upregulation in certain diseases causes fibrosis in different organs and alters signalling pathways of angiogenesis and ECM deposition [2, 34], and PAI‐1(rs1799768) which is involved in proteolysis, in the last phase of the healing process [12].

This study has several limitations. Although the sample of this study is smaller than desirable, due to the difficulty of sample collection, both groups (cases and controls) have a very similar number and a low number of individuals. Significant patient factors in this study, such as sex, lead us to consider the importance of increasing our women's sample including different age subgroups, since in the present study, we have not been able to detect a statistical difference. Furthermore, this study only considered Caucasian individuals, which leads us to believe that including other ethnicities could be very valuable in avoiding extrapolation, overtaking the limitation of the low sample diversity. Finally, the use of PRP was not randomized as it depended on patient acceptance, which could be a bias in the study.

ACL reconstruction is one of the most relevant surgeries in sports medicine, and this study would help to improve the prognosis of the risk of arthrofibrosis in individuals who must undergo this surgical intervention. The development of this prognostic algorithm allows clinicians to know and better understand the weight of genetic variants as well as the impact of the patient variables that surround them, opening the door to the prevention of this condition. Thus, a patient with a high predisposition may need more preoperative physical adaptation or a more justified indication for PRP application. These advances would make it possible to improve the quality of life in the post‐operative period, leading inevitably to more personalized medicine practice.

## CONCLUSION

Genetic alterations involved in inflammation and ECM turnover predispose to the development of arthrofibrosis after ACL reconstruction. Female sex was a risk factor in the development of this condition, while the application of PRP provided a preventive effect The combination of patient and genetic variants of a patient allows the development of a prognostic algorithm for the risk of post‐surgical arthrofibrosis.

## AUTHOR CONTRIBUTIONS

Mikel Sánchez, Diego Delgado, David Celorrio and Jose María Aznar contributed to the conception and design of the study. Mikel Sánchez, Leonor López de Dicastillo, João Espregueira‐Mendes, David Celorrio, Beatriz Aizpurua and Juan Azofra contributed to the provision of study materials and patients. Cristina Jorquera, Izarbe Yarza, Jose María Aznar, Cristina Valente, Renato Andrade and Diego Delgado contributed to the analysis and interpretation of the data. Mikel Sánchez, Diego Delgado, Izarbe Yarza, Cristina Valente, Renato Andrade and Jose María Aznar contributed to the drafting, writing, critical revision and final approval of the article.

## CONFLICT OF INTEREST STATEMENT

Izarbe Yarza is an employee, and Jose María Aznar and David Celorrio are co‐founders of Baigene, which is a Company dedicated to genetic analysis. The remaining authors declare no conflicts of interest.

## ETHICS STATEMENT

Ethical approval for this study was obtained from the Ethics Committee of the Basque Country (UCA‐07/EE/17/FIB).

## Supporting information

Supporting information.

**Figure S1.** X‐ray knee anterior‐posterior (A) and lateral (B) views after anterior cruciate ligament reconstruction.

## Data Availability

The data presented in this study are available within the article. Additional inquiries may be directed to the corresponding author.
