# Supplementary material for: Genetics, sex and the use of platelet‐rich plasma influence the development of arthrofibrosis after anterior cruciate ligament reconstruction
Source: J Exp Orthop. 2025 Jan 28;12(1):e70156. doi: 10.1002/jeo2.70156 (PMC11775413; doi:10.1002/jeo2.70156)
Supplement: Supplementary file 1 — Supporting information. [file JEO2-12-e70156-s002.docx]

| **Table S1.** Genetic polymorphisms included in the study | | | | | |
| --- | --- | --- | --- | --- | --- |
| **Gene** | **Ploymorphism** | **Genotypes** | **Control** | **Cases** | **Position** |
| AQP1 | rs1049305 | C:C | 14 | 5 | chr7:30924207 (GRCh38.p14 |
|  |  | C:G | 18 | 25 |  |
|  |  | G:G | 13 | 14 |  |
| HIF1A | rs11549465 | C:C | 35 | 34 | chr14:61740839 (GRCh38.p14) |
|  |  | C:T | 10 | 9 |  |
|  |  | T:T | 0 | 1 |  |
| GDF5 | rs143383 | C:C | 11 | 6 | chr20:35438203 (GRCh38.p14) |
|  |  | C:T | 23 | 20 |  |
|  |  | T:T | 11 | 18 |  |
| NOS3 | rs1799983 | G:G | 18 | 15 | chr7:150999023 (GRCh38.p14) |
|  |  | G:T | 20 | 25 |  |
|  |  | T:T | 7 | 4 |  |
| TNF | rs1800629 | A:A | 2 | 1 | chr6:31575254 (GRCh38.p14) |
|  |  | A:G | 8 | 13 |  |
|  |  | G:G | 35 | 30 |  |
| ILD6 | rs1800795 | C:C | 4 | 12 | chr7:22727026 (GRCh38.p14) |
|  |  | C:G | 24 | 22 |  |
|  |  | G:G | 17 | 10 |  |
| VEGFA | rs2010963 | C:C | 4 | 7 | chr6:43770613 (GRCh38.p14) |
|  |  | C:G | 22 | 20 |  |
|  |  | G:G | 19 | 17 |  |
| TNC | rs2104772 | A:A | 12 | 18 | chr9:115046506 (GRCh38.p14) |
|  |  | A:T | 22 | 21 |  |
|  |  | T:T | 11 | 5 |  |
| IL6R | rs2228145 | A:A | 16 | 11 | chr1:154454494 (GRCh38.p14) |
|  |  | A:C | 25 | 26 |  |
|  |  | C:C | 4 | 7 |  |
| IGF2 | rs3213221 | C:C | 6 | 7 | chr11:2135814 (GRCh38.p14) |
|  |  | C:G | 17 | 16 |  |
|  |  | G:G | 22 | 21 |  |
| CASP8 | rs3834129 | D:D | 14 | 13 | chr2:201232809D201232814 (GRCh38.p14) |
|  |  | I:D | 21 | 21 |  |
|  |  | I:I | 10 | 10 |  |
| ACE | rs4343 | D:D | 16 | 11 | chr17:63488670 (GRCh38.p14) |
|  |  | I:D | 21 | 29 |  |
|  |  | I:I | 8 | 4 |  |
| SOD2 | rs4880 | A:A | 8 | 12 | chr6:159692840 (GRCh38.p14) |
|  |  | G:A | 20 | 25 |  |
|  |  | G:G | 17 | 7 |  |
| ADRB3 | rs4994 | C:T | 8 | 12 | chr8:37966280 (GRCh38.p14) |
|  |  | T:T | 37 | 32 |  |
| MMP3 | rs679620 | A:A | 8 | 5 | chr11:102842889 (GRCh38.p14) |
|  |  | A:G | 26 | 17 |  |
|  |  | G:G | 11 | 22 |  |
| TP53 | rs1042522 | C:C | 17 | 15 | chr17:7676154 (GRCh38.p14) |
|  |  | C:G | 13 | 20 |  |
|  |  | G:G | 15 | 9 |  |
| TIMP3 | rs5749511 | C:C | 39 | 42 | chr22:32800398 (GRCh38.p14) |
|  |  | C:T | 6 | 2 |  |
| CTGF | rs9493150 | C:C | 5 | 2 | chr6:131952851 (GRCh38.p14) |
|  |  | C:G | 15 | 27 |  |
|  |  | G:G | 25 | 15 |  |
| COL1A1 | rs1800012 | G:G | 23 | 28 | chr17:50200388 (GRCh38.p14) |
|  |  | G:T | 17 | 13 |  |
|  |  | T:T | 5 | 3 |  |
| CRP | rs1800947 | C:C | 25 | 35 | chr1:159713648 (GRCh38.p14) |
|  |  | C:G | 9 | 5 |  |
|  |  | G:G | 11 | 4 |  |
| IL6 | rs1800796 | C:G | 7 | 8 | chr7:22726627 (GRCh38.p14) |
|  |  | G:G | 38 | 36 |  |
| CAT | rs1001179 | A:A | 2 | 2 | chr11:34438684 (GRCh38.p14) |
|  |  | A:G | 20 | 13 |  |
|  |  | G:G | 23 | 29 |  |
| TCF7L2 | rs12243326 | C:C | 1 | 3 | chr10:113029056 (GRCh38.p14) |
|  |  | C:T | 22 | 17 |  |
|  |  | T:T | 22 | 24 |  |
| COL5A1 | rs12722 | C:C | 7 | 11 | chr9:134842570 (GRCh38.p14) |
|  |  | C:T | 21 | 22 |  |
|  |  | T:T | 17 | 11 |  |
| 1q41 (LOC124904518) | rs873549 | A:A | 24 | 21 | chr1:222098425 (GRCh38.p14) |
|  |  | A:G | 15 | 20 |  |
|  |  | G:G | 6 | 3 |  |
| PAID1  (SERPINE1) | rs2227631 | A:A | 14 | 10 | chr7:101126257 (GRCh38.p14) |
|  |  | A:G | 25 | 21 |  |
|  |  | G:G | 6 | 13 |  |
| ADAM33 | rs612709 | A:G | 12 | 10 | chr20:3671560 (GRCh38.p14) |
|  |  | G:G | 33 | 34 |  |
| BMP4 | rs17563 | C:C | 11 | 9 | chr14:53950804 (GRCh38.p14) |
|  |  | C:T | 25 | 23 |  |
|  |  | T:T | 9 | 12 |  |
| NEDD4 | rs8032158 | C:C | 9 | 4 | chr15:55902679 (GRCh38.p14) |
|  |  | C:T | 20 | 16 |  |
|  |  | T:T | 16 | 24 |  |
| BPESC1 | rs940187 | A:A | 5 | 5 | chr3:139122751 (GRCh38.p14) |
|  |  | A:G | 18 | 14 |  |
|  |  | G:G | 22 | 25 |  |
| MMP3 | rs650108 | A:A | 4 | 8 | chr11:102838056 (GRCh38.p14) |
|  |  | A:G | 22 | 21 |  |
|  |  | G:G | 19 | 15 |  |
| SMAD4 | rs12456284 | A:A | 21 | 22 | chr18:51083598 (GRCh38.p14) |
|  |  | A:G | 21 | 18 |  |
|  |  | G:G | 3 | 4 |  |
| TGFDβ1 | rs8110090 | A:A | 40 | 39 | chr19:41339967 (GRCh38.p14) |
|  |  | A:G | 5 | 5 |  |
| PAID1  (SERPINE1) | rs1799768 | D:D | 11 | 8 | chr7:101126425D101126426 (GRCh38.p14) |
|  |  | D:G | 25 | 16 |  |
|  |  | G:G | 9 | 20 |  |
|  |  |  |  |  |  |
