# Supplementary figures and images for: Genetics, sex and the use of platelet‐rich plasma influence the development of arthrofibrosis after anterior cruciate ligament reconstruction
Source: J Exp Orthop. 2025 Jan 28;12(1):e70156. doi: 10.1002/jeo2.70156 (PMC11775413; doi:10.1002/jeo2.70156)

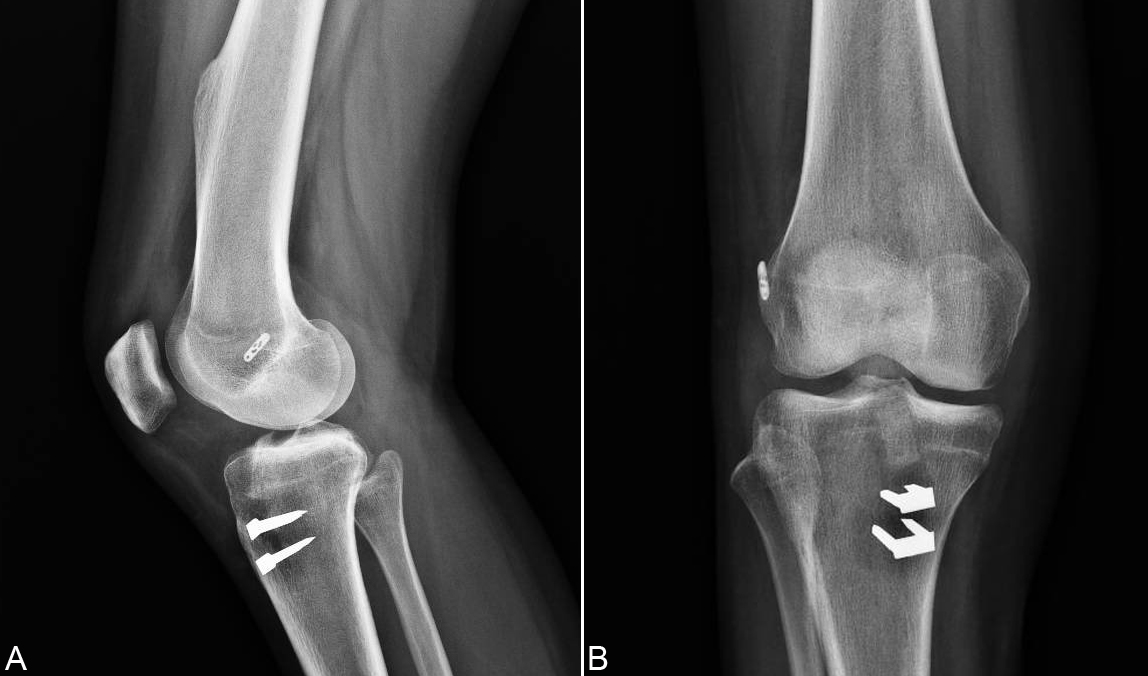

Supplement: Supplementary file 2 — Figure S1. X‐ray knee anterior‐posterior (A) and lateral (B) views after anterior cruciate ligament reconstruction. [file JEO2-12-e70156-s001.jpg]
